# Opioid-induced immunosuppression of brain myeloid cells in SIV-infected rhesus macaques

**DOI:** 10.21203/rs.3.rs-7394731/v1

**Published:** 2025-09-18

**Authors:** Howard Fox, Xiaoke Xu, Meng Niu, Benjamin Lamberty, Katy Emanuel, Shannon Callen, Shilpa Buch, Arpan Acharya, Siddappa Byrareddy

**Affiliations:** University of Nebraska Medical Center; University of Nebraska Medical Center; University of Nebraska Medical Center; University of Nebraska Medical Center; University of Nebraska Medical Center; University of Nebraska Medical Center; University of Nebraska Medical Center; University of Nebraska Medical Center; University of Nebraska Medical Center

## Abstract

Microglia and CNS-associated macrophages (CAMs) are the primary targets of human immunodeficiency virus (HIV-1) in humans and simian immunodeficiency virus (SIV) infection in nonhuman primates, contributing to HIV-associated neurocognitive disorders and establishment of a persistent viral reservoir in the central nervous system (CNS). Despite antiretroviral therapy (ART), neurocognitive and other neurological disorders persist in people with HIV (PWH). Opioid users can suffer exacerbated disease progression and neurological complications. However, the impact of ART-treated infection and opioids on brain myeloid cells, the main targets for HIV/SIV in the brain, remains poorly understood. Using an SIV-infected rhesus macaque model and single-cell multi-omic sequencing, we show that ART promotes the restoration of homeostatic microglial states, while morphine exerts immunosuppressive effects on brain myeloid cells. Consistent with this immunosuppression, in morphine-treated, SIV-infected ART-suppressed macaques we found that myeloid cells exhibited reduced antiviral gene expression, including downregulation of MHC class II and interferon-stimulated genes, as well as decreased activity of AP-1 and ETS transcription factors. Furthermore, through the integration of single-cell data from PWH, we found that homeostatic microglial signatures were also evident in ART-treated PWH, although the microglia from PWH exhibited more activated/inflammatory phenotypes than those from the macaque model. These findings reveal distinct effects of ART and morphine on brain myeloid cell dynamics during SIV infection, indicating potential mechanisms underlying worsened neurocognitive outcomes in opioid-using PWH.

## Introduction

Human immunodeficiency virus (HIV-1) remains a major global public health concern, affecting millions worldwide. According to the latest report from the World Health Organization (WHO), approximately 39.9 million (36.1– 44.6 million) people were living with HIV-1 in 2023^1^, with the highest burden observed in sub-Saharan Africa^2^. Beyond its impact on the immune system, HIV-1 also causes dysfunction in multiple tissues and organs, including the brain. Prior to the introduction of antiretroviral therapy (ART), HIV-1 infection had a high motility rate^3^. However, monotherapy proved ineffective due to the rapid emergence of drug-resistant mutations in the HIV-1 genome, which led to the development of the first combination ART regimen^4^. Despite the success of ART in reducing HIV-1-related mortality and morbidity, HIV-associated neurocognitive disorders persist, affecting around 50% of people with HIV (PWH), albeit with less severe manifestations (PWH)^5^. While the mechanism(s) underlying neurocognitive dysfunction in the efficacious ART era remain unclear, microglia and CNS-associated macrophages (CAMs), key contributors to neuroinflammation, brain injury, and viral reservoirs, are thought to play a critical role^6–9^.

Beyond HIV infection itself, other factors can contribute to neurocognitive disorders in PWH, including but not limited to substance misuse. Injection drug use remains a major risk factor for HIV-1 transmission, accounting for 8% of new infections globally, according to the 2022 UNAIDS report^10^. Among the abused substances, opioids are widely used for pain management in PWH, as well as used illicitly. Notably, PWH are more likely to develop opioid use disorders and associated mental health conditions compared to uninfected individuals^11^. Opioids, including morphine, also have been reported to suppress antiviral gene expression in peripheral immune cells^12^, facilitate HIV entry^13,14^, and impair anti-HIV immune responses^15–17^. Morphine has also been found to enhance inflammatory functions in HIV-infected monocyte-derived macrophages (MDMs) that continue to persist despite ART treatment^18^. However, many of these studies relied on *in vitro* models. Moreover, the effects of morphine on CNS-associated macrophages (CAM) and microglia in the absence of HIV infection remain debated. While some studies suggest that morphine activates myeloid cells^19–22^, others report immunosuppressive and proapoptotic effects^23–28^, highlighting the complexity of opioid–immune interactions.

To shed light on these complexities in a well-controlled *in vivo* experiment, we employed single-nucleus RNA sequencing (snRNA-seq) and single-nucleus ATAC sequencing (snATAC-seq) from the same brain myeloid cells to investigate the effects of morphine in ART-treated simian immunodeficiency virus (SIV)-infected rhesus macaques, a well-established model of HIV infection^29,30^. By leveraging high-throughput sequencing and bioinformatics approaches, we characterized brain myeloid cell populations in four different groups of macaques (uninfected, morphine-exposed, ART-treated SIV-infected, and morphine plus ART-treated SIV-infected macaques). Our findings indicated that ART largely restores brain myeloid cell homeostasis, as phenotypic populations in ART-suppressed SIV infection closely resemble those of uninfected control animals, contrasting with the dysregulation observed in untreated infection^31–33^. Furthermore, we did not detect SIV transcripts in brain myeloid cells in eight ART-treated infected animals (examining over 100,000 cells), reinforcing the effectiveness of ART in controlling brain viral load. Consistent with our previous findings^34^, morphine exposure did not significantly alter the pattern of brain myeloid cell phenotypes.

Despite this homeostatic restoration, transcriptomic and chromatin accessibility profiles revealed patterns of immune activation in brain myeloid cells of ART-treated infected macaques, an effect that was more pronounced in brain myeloid cells from ART-treated PWH. Notably, morphine dependence attenuated the enhanced immune activity of brain myeloid cells in ART-treated SIV infection. A similar immunosuppressive effect of morphine was observed in uninfected animals. While the long-term consequences of morphine-induced immunosuppression in the context of ART remain unclear, our data indicates that morphine and/or ART-treated infection modulate microglial and CAM functions, potentially affecting brain hormonal and neuronal systems and exerting an overall negative impact on the CNS.

## Results

### Chronic morphine use compromises the brain ability to mount effective antiviral responses in the context of ART-suppressed SIV infection.

**Figure 1:**
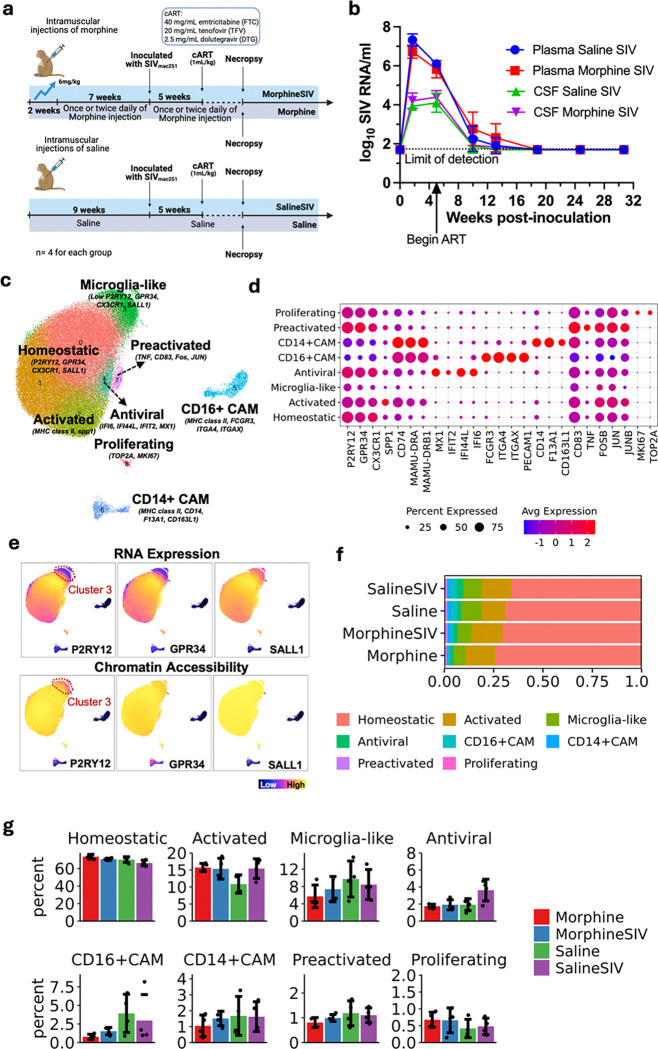
The impact of morphine uses and ART-treated SIV infection on different brain myeloid cell populations. **a.** The schematic illustration of the experimental design. Four groups of animals (n = 4 for each group) with different morphine and infection regimens were involved in this study. **b.** The plasma and CSF viral load for animals in Saline SIV and Morphine SIV groups before and after ART treatment (±SD). **c.** UMAP projection of 105,818 brain myeloid cells from the 16 animals involved in this study. The cells were colored by the cluster information. **d.** Dot plot for selected markers in each cell cluster. **e.** UMAP projection of RNA expression (upper panel) or predicted gene activity (lower panel) of homeostatic microglia core genes, including P2RY12, GPR34, and SALL1. Cluster 3 (Microglia-like) is indicated. Predicted gene activity was calculated using the snATAC-seq data. **f.** Aggregated bar chart of the proportion of brain myeloid cell clusters in each experimental group. **g.** Bar plot of the percentage of each brain myeloid cell phenotype between experimental groups. N=4 for each group.

To understand brain myeloid cell responses in the context of ART-treated SIV infection and morphine administration, we designed four experimental groups [i.e. Saline (uninfected), Saline SIV (ART), Morphine (uninfected), Morphine SIV (Morphine, ART)]) with four rhesus macaques in each group. The two groups of animals received morphine through intramuscular injection for nine weeks (the morphine dose was ramped up to 6 mg/kg over two weeks and maintained for seven weeks, and continued until animals were necropsied), and animals in one of those groups were subsequently inoculated with SIVmac251. Following confirmation of viral infection, peak viremia, and establishment of viral set points, ART was initiated five weeks later to suppress plasma viral loads. Two additional groups of animals followed a similar regimen but with saline injections instead of morphine, again one of the groups received SIVmac251 inoculation and ART treatment (Fig. 1a). The viral loads in plasma and cerebrospinal fluid (CSF) were monitored in animals from the Saline SIV and Morphine SIV groups both before and after initiation of ART (Fig. 1b). Following ART administration, viral loads in both plasma and CSF were reduced to below the limit of detection in both infected groups. No significant difference in viral loads was observed in the context of morphine exposure. Following 6 months of viral suppression with ART, animals were sacrificed and perfused to remove blood-borne cells from the brain, and microglia and CAMs were isolated.

To better understand the transcriptomics and epigenomics of those brain myeloid cells, we performed single-nucleus multiomic sequencing (scMultiome, i.e. snRNA-seq combined with snATAC-seq) on the myeloid cells isolated from the brains of 16 samples. After conducting universal quality control on the sequenced data based on snRNA-seq and snATAC-seq data **(Extended Data Fig. 1a)**, we clustered 106,130 cells using their transcriptomic profiles. The batch effect between individual samples was reduced by running Harmony^35^**(Extended Data Fig. 1b)**. The initial clustering generated 11 cell clusters with only one non-myeloid cell cluster (a lymphocyte cluster) **(Extended Data Fig. 1c)**, which was removed from further analysis.

Further clustering on the remaining 105,818 brain myeloid cells generated nine cell clusters (Fig. 1c) with distinctive transcriptomic characteristics (Fig. 1d **and Supplementary Table 1)**. Clusters 0 and 1 had high expression of homeostatic microglia core genes (e.g. P2RY12, GPR34, and SALL1) without upregulating activation genes, indicating they are homeostatic microglia. Cluster 2 also had high expression of homeostatic microglia core genes, but it was accompanied by increased activation genes (i.e. SPP1 and MHC class II molecules). Cluster 3 had a unique transcriptomic profile, which did not have high expression of either homeostatic microglia markers or activation markers. However, the snATAC-seq analysis showed that the downregulation of homeostatic microglia core genes in this cluster was only at the transcriptomic level (Fig. 1e). Chromatin regions of those homeostatic genes were present in cluster 3, and the accessibility in this cluster was similar to that in clusters 0, 1, and 2 **(Extended Data Fig. 1d)**. Given this, we annotated the cells in this cluster as Microglia-like cells. Cluster 4 was characterized by the high expression of interferon-inducible proteins, suggesting its enhanced antiviral ability, and was denoted as such. Clusters 5 and 6 were CAM clusters with relatively higher expression of MHC class II genes and lower expression of homeostatic microglia core genes than other myeloid cell clusters. Additionally, clusters 5 and 6 were separated from the primary microglial population on the UMAP (Fig. 1c), indicating their different transcriptomic profiles. Given the high expression of CD16 and CD14 in cluster 5 and cluster 6, they were classified as different CAM phenotypes like that circulating non-classical (CD16^+^) and classical (CD14^+^) monocytes, respectively. Cluster 7 upregulated CD83, TNF, FOS, and JUN but without upregulation of activation genes (Fig. 1d **and Supplementary Table 1)**, and were classified as preactivated microglia^36^. Finally, the high expression of homeostatic microglia core genes and proliferating markers in cluster 8 indicated these were proliferating microglia. The pathway analysis based on the DEGs found in each brain myeloid cell cluster also confirmed this **(Extended Data Fig. 1e and Supplementary Table 2)**. The DEGs found in Cluster 4 (antiviral microglia) revealed enrichment with pathways related to virus responses, which were associated with many other antiviral genes other than interferon-related genes **(Extended Data Fig. 1f)**.

Comparison of myeloid cell composition across treatment groups revealed minimal comprehensive differences (Fig. 1f), with homeostatic microglia remaining the dominant population. To capture slight changes, we compared the percentage of cells between treatment groups within each cluster (Fig. 1g) and performed cell type composition analysis using propeller method^37^ implemented by speckle R package. Given the relatively small number of animals involved in each group, the statistics did not show significance between groups for any of the cell cluster (**Supplementary Table 3**). However, the comparison showed that antiviral microglia were increased in the Saline SIV group compared to the other three groups with relatively smaller FDR. In particular, in the Saline SIV group 3.7% of the myeloid cells were of the antiviral phenotype, compared to 1.9% in the Morphine-SIV group, which was similar to the levels in the uninfected Saline (1.9%) and Morphine (1.7%) groups. Given their expression of interferon-responsive and other antiviral genes, this reduction suggests that morphine may impair the ability to keep the virus suppressed during ART treatment. Additionally, we observed a decrease of CD16+ CAM in morphine-treated groups compared to non-morphine-treated groups (FDR for Morphine vs Saline was ~ 0.07), and an increase of activated microglia in the Saline SIV, Morphine, and Morphine SIV groups compared to the Saline controls with relatively small FDR value. Although the overall shifts in myeloid composition were subtle, the Saline SIV group displayed reduced homeostatic and increased activated and antiviral microglia, indicating a relatively heightened activation state in response to SIV infection compared to the Morphine SIV group.

### Morphine modestly suppresses immune activity in brain-resident myeloid cells of SIV-infected rhesus macaques treated with ART.

**Figure 2:**
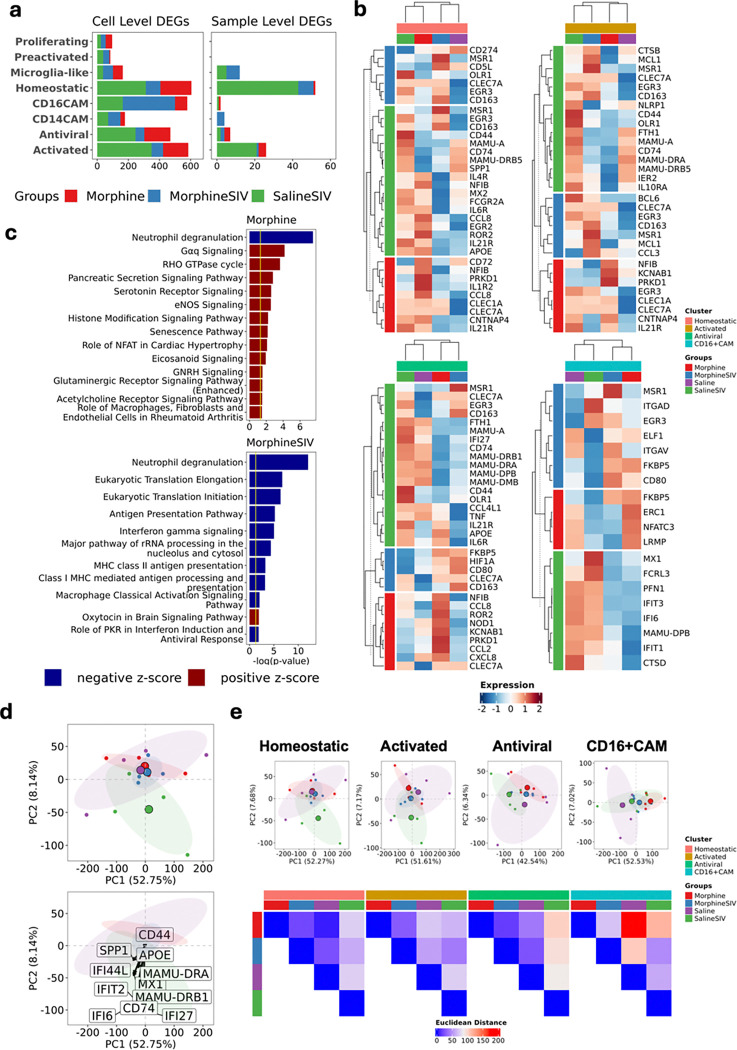
Assessment of the effect of morphine and ART-treated SIV infection on the transcriptomic profiles of brain myeloid cells. **a.** An aggregated bar chart shows the number of cell-level (based on the single cell data) and sample-level DEGs (based on pseudobulk data) for each treatment group and myeloid cell cluster. **b.** The expression of cell-level DEGs in four experimental groups and key myeloid cell clusters. The row annotations indicate the group where DEGs were found. The normalized RNA counts were scaled for plotting. For the DEGs showed in **a** and **b**, they were identified between the treatment (SalineSIV, MorphineSIV, or Morphine) groups and the control (Saline) group. For single-cell data, the Wilcoxon rank sum test was used, and for pseudobulk data, a negative binomial model with the Wald test implemented in DESeq2 was used. The cut-off for detecting DEGs was set as FDR ≤ 0.05 or adjusted p-value ≤ 0.05 and log_2_FC ≥ 0.25. **c.** The cell-level DEGs (FDR ≤ 0.05 and absolute log_2_FC ≥ 0.5) found for MorphineSIV and Morphine groups (SalineSIV and Saline was set as control for each of comparison) were enriched into pathways through Ingenuity Pathway Analysis (IPA). The IPA cut-off for the Morphine group was set as −log(p-value) > 1.3 and absolute z score > 2.5, and for the MorphineSIV group was set as −log(p-value) > 1.3 and absolute z score > 2.3. The upregulated pathways have a positive z-score, and the downregulated pathways have a negative z-score. **d.** PCA for 16 samples from four different experimental groups. All brain myeloid cells were used for pseudobulking, and top 5000 highly variable genes were used for PCA. Selected microglia/macrophage activation genes were shown on the lower panel. **e.** PCA for each key brian myeloid cell cluster (upper panel). The brain myeloid cells in each cluster were used for pseudobulking, and and top 5000 highly variable genes were used for PCA. The Euclidean distance was then calculated between groups based on PCA (lower panel).

To investigate the distinct effects of SIV infection and/or morphine exposure on specific brain myeloid cell populations, we performed differential gene expression analyses using both single-cell (cell-level) and pseudobulk (sample-level) transcriptomic data **(Supplementary Tables 4 and 5)**. Comparisons were made between each of the three experimental groups (Saline SIV, Morphine, and Morphine SIV) and the Saline control group. The largest number of differentially expressed genes (DEGs) was identified between the Saline SIV and Saline groups, particularly within the homeostatic microglia, antiviral microglia, activated microglia, and CD16⁺ CAM clusters (Fig. 2a). As a result, subsequent analyses focused on these four key microglial or CAM subsets. At the cell level (Fig. 2b), DEGs in the Saline SIV group showed marked upregulation of MHC class II molecules and other activation-related genes, particularly within microglial clusters. Notably, the expression of these genes was further reduced in the Morphine and Morphine SIV groups. In the CD16⁺ CAM cluster, the Morphine and Morphine SIV groups exhibited significant downregulation of interferon-related genes compared to both Saline and Saline SIV groups. Although the Morphine SIV group showed higher expression of certain activation genes than the Morphine group, overall expression remained lower relative to the Saline SIV group. To further assess morphine’s immunosuppressive effects, we compared Morphine vs. Saline and Morphine SIV vs. Saline SIV across all brain myeloid cells **(Extended Data Fig. 2a–2b)** and performed pathway enrichment analysis on the DEGs. In both uninfected and ART-treated infected conditions, the neutrophil degranulation pathway was suppressed. Additional immune-related pathways, including antigen presentation, interferon-gamma signaling, and classical macrophage activation, were also significantly downregulated in the Morphine SIV group (Fig. 2c), suggesting that morphine exerts a stronger immunosuppressive effect during ART-treated SIV infection.

Interestingly, CLEC7A expression, found to be upregulated in disease-associated microglia in a variety of conditions, an established regulator of synaptic phagocytosis^38,39^ was significantly upregulated in Saline SIV, Morphine SIV, and Morphine groups within numerous microglial clusters. CLEC7A is known to regulator of synaptic phagocytosis^38,39^ This suggests that CLEC7A may be modulated by both ART-treated SIV infection and morphine exposure. CLEC7A was also significantly upregulated across multiple brain myeloid clusters at the sample level **(Extended Data Fig. 2c–2e)**.

In contrast to CLEC7A, several DEGs identified at the sample level exhibited inconsistent expression across individual animals. For instance, ADORA2B, an inflammation-associated receptor, was significantly upregulated in the Saline SIV group, especially in homeostatic and activated microglia, but this upregulation was observed in only two of the four animals in that group **(Extended Data Fig. 2d–2f)**, highlighting notable inter-animal variability. Additionally, downregulation of microglial activation genes detected at the cell level in the Morphine and Morphine SIV groups was not evident in the sample-level analysis, suggesting that morphine-induced suppression may be more subtle and heterogeneous across individuals.

To further assess the impact of SIV and morphine on the global transcriptomic landscape, we performed principal component analysis (PCA) on the top 5,000 highly variable genes identified from pseudobulked brain myeloid cell data. This gene set captured most of the transcriptomic variance, with no appreciable gain from including more genes. In the PCA plot, Saline SIV samples separated from the other groups along the first two principal components, primarily driven by genes involved in MHC class II presentation, antiviral responses, and inflammatory activation (Fig. 2d). At the cluster level, the Euclidean distances between Saline SIV and the other groups were notably higher, particularly in the antiviral microglial cluster, while the Morphine and Morphine SIV groups exhibited smaller intergroup distances (Fig. 2e), further supporting the immunosuppressive effect of morphine. Nevertheless, consistent with the DEG analysis, intra-group variability was observed among individual samples in the PCA.

In summary, our findings suggest that morphine attenuates microglial activation under both uninfected and ART-treated SIV-infected conditions. However, the variability among animals implies that these effects are moderate and influenced by inter-individual differences. While larger sample sizes may be needed to detect potential statistically significant effects, the overall transcriptomic patterns support a role for morphine in dampening immune activation in the brain during SIV infection.

**Figure 3:**
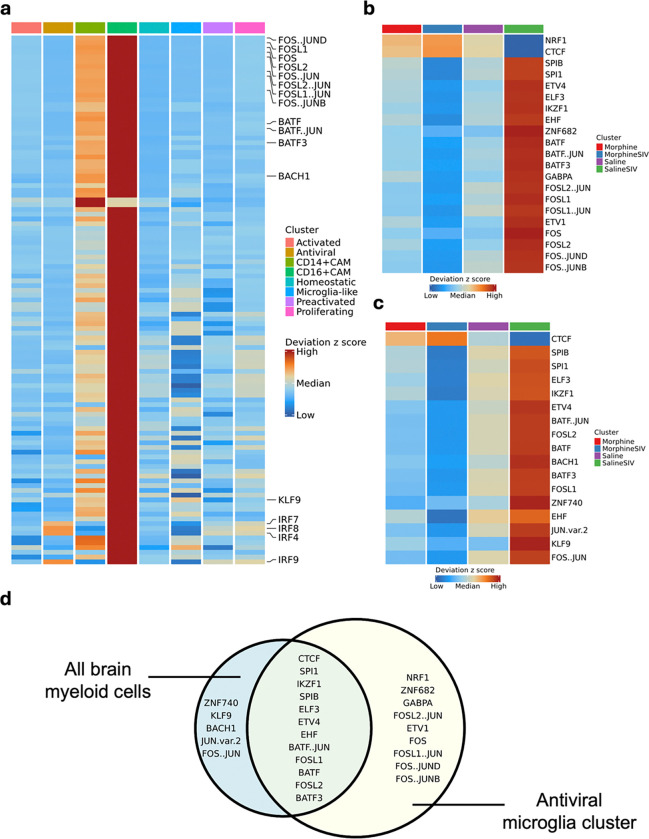
Differential transcription factor (TF) enrichment in brain myeloid cells across treatment groups. **a.** Heatmap for motif deviation score of TFs with an FDR ≤ 0.05 and mean difference ≥ 1 over different cell clusters.**b.** Heatmap for motif deviations of TFs with an FDR ≤ 0.05 and mean difference ≥ 0.5 over different treatment groups in antiviral microglia cluster. **c.** Heatmap for motif deviations of TFs with an FDR ≤ 0.05 and mean difference ≥ 0.5 over different treatment groups in all brain myeloid cell clusters. **d.** Venn diagram for common or different TFs with an FDR ≤ 0.05 and mean difference ≥ 0.5 between antiviral microglia cluster and all brain myeloid cell clusters.

To further investigate the epigenomic impact of morphine administration and ART-treated SIV infection, we analyzed transcription factor (TF) enrichment analysis across distinct brain myeloid cell clusters and treatment conditions. Notably, the TF enrichment profiles in CAM clusters were markedly distinct from those observed in microglial clusters, whereas the differences among the various microglial subpopulations were relatively subtle (Fig. 3a). However, antiviral microglial clusters exhibited substantially higher enrichment of IRF4, IRF7, IRF8, and IRF9 compared to other microglial clusters. Given the pivotal roles of IRF7, IRF8, and IRF9 in interferon signaling^40–42^, these findings underscored the enhanced interferon- producing capacity of antiviral microglia. As previously noted, morphine administration was associated with a reduction in the antiviral microglial population and downregulation of activation-associated genes within this cluster. To further elucidate the regulatory landscape underlying this suppression, we compared TF enrichment within the antiviral microglial cluster across treatment groups (Fig. 3b). As expected, the majority of significantly enriched TFs were observed in the Saline SIV group. An exception to this trend included NRF1 and CTCF, which demonstrated significantly higher enrichment in morphine-treated groups. Notably, based on our defined statistical threshold, no TFs in the IRF family exhibited significant differences between treatment conditions. Nevertheless, a number of TFs involved in regulating immune and inflammatory responses in hematopoietic cells, particularly members of the AP-1 and ETS families, were significantly upregulated in the Saline SIV group. This increased enrichment was evident not only in antiviral microglial clusters but also in broader brain myeloid populations (Fig. 3c and Fig. 3d). Conversely, enrichment of TFs in AP-1 and ETS families was consistently reduced in Morphine SIV groups, further supporting the immunosuppressive effect of morphine on brain-resident myeloid cells in ART-treated SIV infection.

### Gene co-expression network analysis reveals distinct myeloid cell responses to ART-treated SIV infection and morphine use.

**Figure 4:**
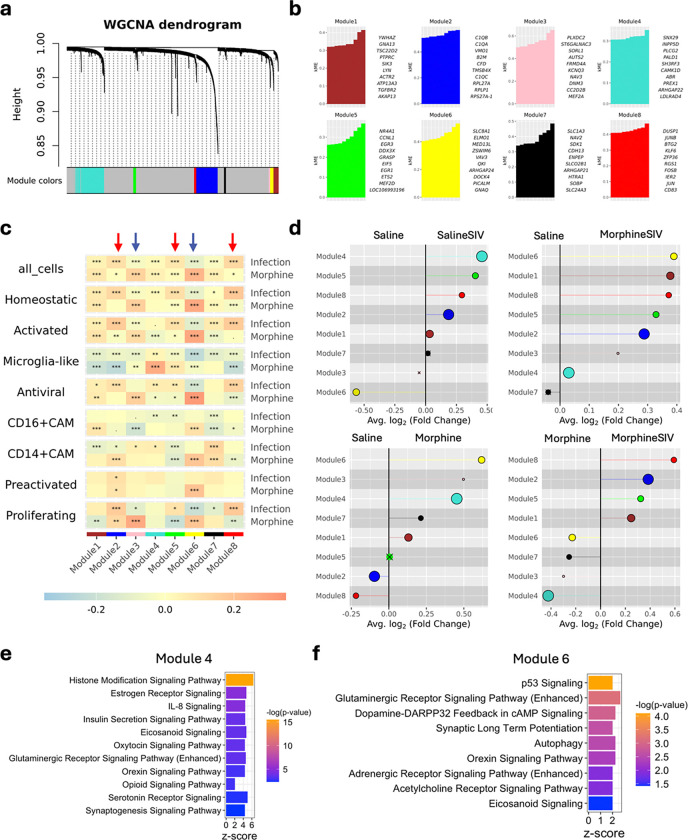
The distinctive gene modules for morphine use and/or ART-treated SIV infection revealed by WGCNA. **a.** WGCNA dendrogram shows the different co-expression modules identified for brain myeloid cells from all treatment groups. **b.** The top 10 highest connected genes (hub genes) for each identified module. The genes were ranked by kME (module eigengene-based connectivity). **c.** The module-trait correlation heatmap shows the correlation between infection/morphine and each module. The modules showing a relatively stronger correlation with infection or morphine were indicated by red or blue arrows. The heatmap was colored by correlation coefficient and the asterisks labeled on the heatmap showed the FDR-corrected p-values. “.” 0.05 < p ≤ 0.1, * 0.01 < p ≤ 0.05, ** 0.001 < p ≤ 0.01, *** p ≤ 0.001. **d.** Lollipop plot shows the up- or down-regulated modules in two treatment groups. The groups used for each comparison were labeled on the top of the plot. The modules with negative fold-change were upregulated in the group labeled on the left, and the modules with positive fold-change were upregulated in the group labeled on the right. The size of each dot corresponds to the number of genes in that module. An “X” is placed over each point that did not reach statistical significance. **e, f.** The selected enriched terms for the hub genes in **e,** module 4 and **f,** module 6. The pathway analysis was performed with Ingenuity Pathway Analysis (IPA) and the analysis was based on eigengene-based connectivity (kME) calculated for each hub gene. The pathways with −log(p-value) > 1.3 and z-score ≥ 2 were considered as significantly upregulated pathways.

To further uncover gene co-expression patterns and functional differences across different brain myeloid cell clusters and treatment groups, we performed single-cell Weighted Gene Co-expression Network Analysis (WGCNA) by using hdWGCNA R package^43^. We identified eight gene modules across all brain myeloid cell clusters from four experimental groups (Fig. 4a). The core genes exhibited the strongest intramodular connectivity, providing insights into the biological functions of each module (Fig. 4b **and Supplementary Table 6)**. Notably, modules 2, 5, and 8 appeared to be associated with myeloid cell activation. For instance, the top 10 hub genes in module 2 included C1QA, C1QB, C1QC, and CFD, which are involved in the complement pathway (Fig. 4b). Additionally, several key microglial and macrophage markers (CSF1R, CST3, APOE, FTL, AIF1, P2RY12, and CX3CR1) exhibited strong connectivity to this module, suggesting its relevance to diverse brain myeloid cell functions **(Supplementary Table 6)**. Module 5 appeared to be involved in myeloid cell activation and inflammation, as it contained hub genes from the Early Growth Response (EGR) family, along with CD163, IL1B, CXCL8, and CD80. Module 8 featured hub genes from the activator protein-1 (AP-1) transcription factor family (JUNB, FOSB, JUN, FOS, and JUND), as well as markers of alternative macrophage activation (CD83) and macrophage scavenger receptors (MSR1). Pathway analysis confirmed that hub genes in these three modules were linked to neuroinflammation signaling pathways **(Extended Data Fig. 3a and Supplementary Table 7)**. Distinct biological processes were found to be enriched in each of these modules. Module 2 was associated with pathogen recognition, phagocytosis, and antigen presentation, module 5 was enriched for cytokine and fibrosis-related pathways, and module 8 was linked to oxidative stress and autophagy.

When analyzing module expression across cell clusters **(Extended Data Fig. 3b)**, module 2 showed higher expression in CD14+ CAM and antiviral microglial clusters, while modules 5 and 8 were predominantly expressed in the preactivated microglial cluster. In terms of treatment groups **(Extended Data Fig. 3c)**, module 2 was upregulated in the Saline SIV and Morphine SIV groups, suggesting its specificity to SIV infection. In contrast, module 6 was highly expressed in the Morphine SIV and Morphine groups, indicating its association with morphine exposure. Interestingly, modules 3 and 4 showed higher expression in the Morphine group but not in the Morphine SIV group. Module-trait correlation analysis further supported these findings (Fig. 4c). Modules 2, 5, and 8, which were linked to microglial/macrophage activation, positively correlated with infection but showed no correlation with morphine use. In contrast, modules 3 and 6 displayed positive correlations with morphine use and either no or negative correlation with infection, suggesting that morphine use does not promote microglial/macrophage activation.

To further explore the divergence of gene modules across experimental groups, we performed differential module eigengene (DME) analysis between each pair of treatment groups (Fig. 3c **and Extended Data Fig. 3d)**. Consistent with the module-trait correlation analysis, modules 2, 5, and 8 were significantly upregulated in the infected groups (Saline SIV and Morphine SIV) compared to the uninfected groups (Saline and Morphine). Notably, when comparing Saline SIV to Morphine SIV **(Extended Data Fig. 3d)**, modules 2 and 8 were elevated in Morphine SIV, while module 5 was higher in Saline SIV, although the average log_2_ fold change was small. Intriguingly, module 4 was consistently upregulated in both the Saline SIV and Morphine groups. This module also exhibited a positive correlation with both infection and morphine use, suggesting that it may be modulated by both factors. Pathway enrichment analysis revealed that the lowest p-value and the highest z-score change was for histone modification signaling within module 4 (Fig. 4e). This finding suggests that ART-treated SIV infection and morphine use may induce epigenetic changes in brain myeloid cells, driving gene expression and phenotypic changes. Indeed, proper control of histone modifications is key in microglia development and homeostasis and is altered in neurodegenerative disease^44^. Additionally, several signaling-related pathways including estrogen receptor signaling, insulin secretion signaling, and opioid signaling were enriched in module 4. Many endocrine hormone receptors are known to be expressed on microglia where they modulate pro-inflammatory or anti-inflammatory responses^45–47^. Notably, the enrichment of opioid signaling further supports the observed positive correlation between module 4 and morphine exposure. A different module, module 6, was significantly upregulated in the Morphine SIV group relative to Saline SIV, with an average log_2_ fold change of approximately 1. This module was also significantly elevated in the Morphine and Morphine SIV groups compared to the Saline group. Moreover, when comparing Morphine to Morphine SIV (Fig. 4c), module 6 remained significantly higher in the Morphine group, suggesting a robust influence of morphine exposure on the genes within this module. Module 6 shows significant enrichment in the p53 signaling pathway. In PWH with neurocognitive disorders, increased p53 expression was found in microglia, and p53 expression in microglia is associated with a proinflammatory phenotype^48,49^.

In summary, our results suggest that ART-treated SIV infection drives direct myeloid cell activation and immune responses that are not associated with or are negatively correlated to morphine use indicating that each condition uniquely shapes myeloid cell function in the brain. However, morphine exposure also could affect the function and activation of brain myeloid cells which might be through indirect signaling (e.g. through endocrine receptors).

### More activated brain myeloid cells were observed for ART-treated HIV infection in humans compared to ART-treated SIV infection in the rhesus macaque.

To assess the translatability of our findings to humans, we next compared the results of this study with scRNA-seq data from ART-treated viremia-suppressed PWH, as well as other single-cell studies on microglia isolated from humans. To perform this comparison, we integrated both human and rhesus macaque datasets to enable cross-species analysis. Integration of rhesus macaque and human scRNA-seq datasets can be achieved using either general batch effect correction methods or approaches specifically designed for cross-species integration. Based on recent benchmarking studies^50,51^, we selected four batch correction methods, Seurat CCA^52,53^, scVI^54^, scANVI^55^, and scGen^56^, and one cross-species integration method, SATURN^57^, for comparative evaluation. These methods were tested using scRNA-seq data from three uninfected macaques, three ART-treated SIV-infected macaques, and three ART-treated HIV-infected individuals, as outlined in **Extended Data Fig. 4a**.

For general batch effect removal, consistent gene symbols are a prerequisite. Therefore, we evaluated three strategies for orthologous gene mapping: a custom conversion database (see Methods), the orthogene R package using HomoloGene, and gene orthologs built by GeneOrthology R package. Method performance was assessed using integration metrics from the scIB package^58^. All four batch correction methods significantly mitigated batch effects between human and macaque samples **(Extended Data Fig. 4b and Extended Data Fig. 4c)**, with minimal variation arising from the choice of gene conversion database. Among them, scGen with a custom gene conversion database demonstrated superior performance by effectively preserving biological signals across species. Conversely, while SATURN preserved cellular identity, it was less effective in removing batch effects **(Extended Data Fig. 4d)**. Based on these results, we selected scGen with the custom gene conversion database for subsequent integration of a larger dataset.

We then expanded the dataset by including scRNA-seq data from 23 human brain samples: three from ART-treated PWH^59^, one from control (without CNS disorders), and 19 from HIV-negative individuals with various CNS disorders^60^ **(Supplementary Table 8)**. We incorporated all 16 macaque samples from this study. Additionally, three samples from untreated acute SIV infection condition^31^, and three from untreated chronic SIV infection condition ^33^ were included, totaling 22 macaque samples **(Supplementary Table 8)**.

**Figure 5:**
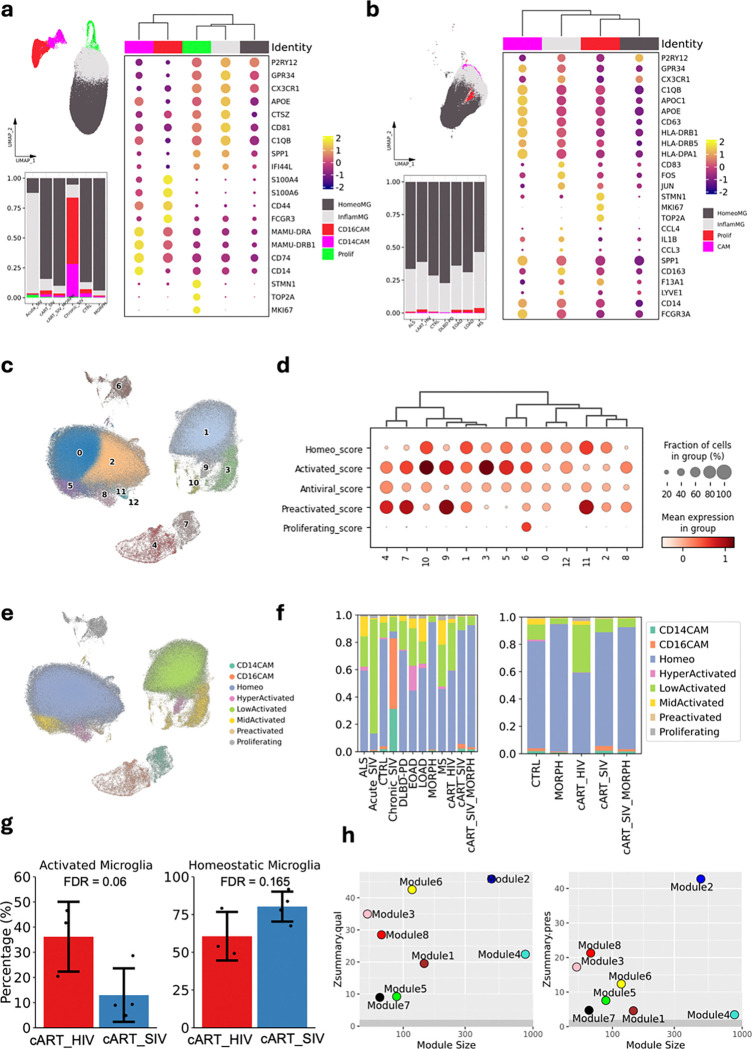
Integrated analysis of microglial subtypes across human and rhesus macaque brain samples. **a.** Analysis for brain myeloid cells from 22 rhesus macaque samples from animals with acute SIV infection (Acute_SIV), chronic SIV infection (Chronic_SIV), ART-treated SIV infection (ART_SIV), ART-treated SIV infection with morphine administration (cART_SIV_MORPH), no infection with morphine administration (MORPH), and control (CTRL). **b.** Analysis for brain myeloid cells from 23 human samples from subjects with ART-treated HIV infection (cART_HIV), Amyotrophic Lateral Sclerosis (ALS), Dementia with Lewy bodies-Parkinson’s disease (DLBD-PD), Early-onset Alzheimer’s disease (EOAD), Late-onset Alzheimer’s disease (LOAD), Multiple sclerosis (MS), and control (CTRL). **c.** UMAP plotting of cells and clusters from an integrated dataset. **d.** Dot plot shows the homeostatic, activated, antiviral, preactivated, and proliferating scores for each cluster. **e.** UMAP plotting of annotated cells from the integrated dataset. **f.** Composition of different brain myeloid cell phenotypes in different groups of samples involved in integrated data. **g.** Comparison of the percentage of activated microglia (sum of low-, mid-, and hyper-activated microglia) and homeostatic microglia between animals in cART_HIV and cART_SIV groups. The statistic test in propeller for cell type composition analysis was implemented, and the FDR was labeled. **h.** The Z-summary stats of module perturbation tests for data from ART-treated HIV samples. The modules were identified using macaque data and projected to data from cART-treated HIV samples. Modules with Z < 2 are unpreserved, modules with 10 > Z >2 are moderately preserved, and modules with Z > 10 are highly preserved in data from ART-treated HIV samples.

Prior to integration, we independently performed unsupervised clustering on the human and macaque datasets to define broad categories of brain myeloid cells. In the macaque dataset, we identified five major populations: homeostatic microglia, inflammatory microglia, proliferating microglia, CD14^+^CAM, and CD16^+^CAM (Fig. 5a). As previously observed, most brain myeloid cells in the Morphine (MORPH), Morphine SIV (ART_SIV_MORPH), Saline (CTRL), and SalineSIV (ART_SIV) groups were homeostatic microglia. In contrast, acute SIV infection was associated with increased inflammatory microglia, whereas chronic infection showed a predominance of cells classified as CAMs, indicating again that ART contributes to the restoration of microglial homeostasis. In the human dataset, we similarly identified homeostatic, inflammatory, and proliferating microglia. However, CD14^+^ and CD16^+^ CAMs formed a single cluster (Fig. 5b). Cell type composition was largely consistent across human individuals analyzed.

Following integration using scGen, batch effects were substantially reduced, while most biological variability was retained. Clustering of the integrated dataset yielded 13 distinct clusters (Fig. 5c). Clusters 4 and 7, corresponding to CD16^+^ and CD14^+^ CAMs, respectively, were exclusively derived from macaque samples. The remaining clusters represented microglial subtypes. Using marker gene-based scoring, we annotated each cluster according to homeostatic, activated (subdivided into low-, mid-, and hyper-activated based on the homeostatic and activated scores), antiviral, preactivated, and proliferative phenotypes (Fig. 5d). Clusters 0 and 2 were homeostatic microglia, while clusters 1 and 12 were classified as low-activated microglia. Clusters 3, 5, and 10 exhibited features of mid-activated microglia, and clusters 8 and 9 were identified as hyper-activated microglia. Cluster 6 was defined as proliferating, and cluster 11 as preactivated microglia (Fig. 5e). None of the clusters had a distinctly elevated antiviral score, with the hyper-activated microglia cluster having a relatively lower antiviral score compared to other clusters.

Analysis of phenotype distribution across samples (Fig. 5f) revealed enrichment of low-, mid-, and hyper-activated microglia in human samples with neurodegenerative disorders. In ART-treated HIV samples, 34.8% of cells were low-activated microglia, and 2.5% of cells were mid-activated microglia, with virtually no hyper-activated microglia. In macaques, the cells from the acute SIV infection condition were dominated by low-activated microglia (~ 83.4%), while those from chronic SIV infection condition showed a predominance of cells classified as CAMs in this joint macaque-human classification. Among the four experimental groups involved in this study, homeostatic microglia were most prevalent (~ 80%–90%), with morphine-treated animals exhibiting the lowest proportion of activated phenotypes (< 5%). Notably, the mid- and hyper-activated microglia detected in the CTRL group (the four uninfected untreated macaques and the one human sample) were primarily derived from the single human sample. The ART-treated SIV-infected macaques exhibited a lower proportion of activated microglia, and a higher proportion of homeostatic microglia compared to ART-treated HIV-infected individuals. Although they did not reach statistical significance, the FDR for activated microglia was 0.06 (Fig. 5g). Perhaps related to this is that we did not find any cells expressing SIV RNA in the examination of 105,818 brain myeloid cells from ART-suppressed macaques, whereas in the study of ART-suppressed PWH, 107 out of 25,091 brain myeloid cells expressed HIV RNA (0.43%). This difference is highly significant (two proportion z-test, z-score −22.07, p<0.0001). Morphine treatment further reduced microglial activation in the macaques. Nevertheless, when compared to other neurocognitive disorders, microglial activation in ART-treated HIV infection appeared relatively mild, primarily involving low- and mid-activated phenotypes.

To investigate the transcriptomic similarities and differences between ART-treated HIV and ART-treated SIV infections, we performed a module preservation analysis using gene co-expression modules previously identified from 16 macaque samples (Fig. 4a and Fig. 4b). Among the modules associated with myeloid cell activation and inflammation that were positively correlated with ART-treated SIV infection (i.e. modules 2, 5, and 8), we observed strong preservation of modules 2 and 8 in ART-treated HIV infection (preservation z-score > 10), while module 5 showed moderate preservation (2 < preservation z-score < 10) (Fig. 5h). Based on the functional pathways enriched in the modules 2 and 8 **(Extended Data Fig. 3a)**, including phagocytosis, pathogen recognition, and oxidative stress, our findings suggest that these biological processes may play critical roles in the context of ART-treated HIV/SIV infection.

## Discussion

By performing single-nucleus multi-omic sequencing on brain myeloid cells from 16 rhesus macaques across four treatment groups, defined by the presence or absence of ART-suppressed SIV infection and morphine administration, we identified eight transcriptionally distinct brain myeloid cell clusters. Although overall cell-type composition did not differ significantly among the groups, we observed a relative increase in homeostatic microglia and a decrease in antiviral microglia in morphine-treated animals compared to the Saline SIV group. Transcriptomic profiling further revealed that multiple activation-related genes and pathways were significantly downregulated in the Morphine and Morphine SIV groups relative to the Saline and Saline SIV groups. Chromatin accessibility analysis showed that transcription factors belonging to the AP-1 and ETS families, which are typically enriched in activated myeloid cells, were markedly less accessible in morphine-treated animals. These motifs were most enriched in the Saline SIV group, followed by the Saline group. Studies found that JUNB in AP-1 family is a potential central regulator for macrophages’ activation^61^ and SPI1 (PU.1) and ELF3 in ETS family play critical roles in myeloid cells’ proinflammatory responses^62,63^. In addition, single-cell gene co-expression network analysis indicated that morphine exposure was either negatively correlated or not correlated with gene modules enriched for immune activation and inflammatory responses.

Despite the absence of overt transcriptional or chromatin-level signatures of activation, our findings suggest that morphine may nonetheless affect brain myeloid cell function, potentially via various signaling pathways. Overall, the data support an immunosuppressive effect of morphine on brain myeloid cells, evident under both uninfected and ART-treated SIV-infected conditions. However, these conclusions are tempered by limitations inherent to the small sample size and inter-individual variability among subjects.

To address these limitations and to explore potential cross-species patterns, we expanded our analysis for brain myeloid cells by integrating single-nucleus RNA-seq data from six additional rhesus macaque brain samples, encompassing a range of SIV infection statuses for a total of 22 rhesus macaque samples, and 23 human brain samples including three from PWH receiving ART, one with no known neurological disorder, and 19 with non-HIV-related neurocognitive disorders. Comparative analysis revealed minimal enrichment of hyperactivated microglial populations in both ART-treated HIV and SIV groups relative to other neurocognitive disorders. Nevertheless, ART-treated PWH displayed a higher proportion of low- and mid-activated microglia and fewer homeostatic microglia compared to ART-treated SIV-infected macaques. This discrepancy may be explained by several factors. First, in human cohorts, ART is often discontinued at the end of life, although subjects included in our study had received treatment for at least two weeks prior to death^59^. Other plausible contributors include reduced ART adherence in human populations, greater variability in treatment regimens, and increased exposure to co-morbid risk factors, such as substance abuse, that exacerbate neuroinflammation and contribute to neurocognitive disorders. These findings suggest that in the current ART era, persistent neurocognitive disorders in PWH may be more closely linked to inconsistent treatment adherence and additional co-morbid exposures than to treatment-resistant active viral replication alone.

Given the known impact of substance use on neurological deficits, we specifically investigated the effects of morphine, a commonly abused opioid, on brain myeloid cells in the context of ART-treated SIV infection. In a prior study using a similar morphine and ART treatment regimen, viral analyses on microglia and CAMs showed low levels of cell-associated viral DNA and RNA, with no significant differences between the morphine and saline groups. However, sensitive assays such as the macrophage quantitative viral outgrowth assay and the intact proviral DNA assay detected significantly higher levels of replication-competent virus in morphine-treated animals^64^.

While the precise mechanisms by which opioids modulate SIV/HIV persistence in the CNS remain incompletely understood, our findings indicate that morphine induces an immunosuppressive shift in brain myeloid cell states. Although this shift was modest in magnitude, it could lead to clinically meaningful effects over time, particularly in the setting of chronic opioid use and ART. Despite previous reports showing that morphine may enhance viral replication in microglia via activation of the mu-opioid receptor (MOR)^65,66^, we did not detect SIV-infected cells (containing either SIV RNA or DNA) in morphine-treated or saline-treated ART-treated SIV-infected animals, consistent with our prior studies in ART-suppressed SIV-infected macaques. Furthermore, MOR expression and chromatin accessibility were largely unchanged in morphine-treated animals. These results may reflect the limited sensitivity of snRNA-seq and snATAC-seq technologies, the viral suppression achieved through concurrent ART administration, and the relatively moderate and progressively titrated morphine dosing used in our model, which contrasts with the higher and more erratic patterns of use typically seen in human substance abuse.

In future studies, integrating larger single-cell datasets from both PWH and PWoH, alongside those from non-human primate models, will be critical for elucidating conserved and species-specific mechanisms driving neurocognitive and other CNS deficits. In addition, regional differences of gene expression phenotypes in brain myeloid cells have been found^36^, and regional effects of SIV/HIV and opioids on these cells can be examined. We note that this study was performed as part of the single-cell opioid responses in the context of HIV (SCORCH) consortium, in which such data from human and animal models, including non-human primate, rat and mouse, will be obtained^67^. Such comparative approaches will enhance translational relevance and improve the fidelity with which animal models can inform our clinical understanding and treatment strategies for these disorders.

## Materials And Methods

### Animals

Sixteen adult male rhesus macaques **(Supplementary Table 9)** were used in this study. All animals tested negative for the indicated viral pathogens: SIV, SRV, STLV1, Herpes B-virus, and measles; and bacterial pathogens: salmonella, shigella, campylobacter, yersinia, and vibrio. The macaques were housed following the Animal Welfare Act and the Guide for the Care and Use of Laboratory Animals at the Department of Comparative Medicine, University of Nebraska Medical Center (UNMC). The primate facility at UNMC is accredited by the American Association for Accreditation of Laboratory Animal Care International. The study was reviewed and approved by the UNMC Institutional Animal Care and Use Committee (IACUC) under protocol 16–073-FC. Animals were maintained in a temperature-controlled indoor environment (23 ± 2° C) with a 12-hour light/dark cycle. They were fed a Teklad Global 25% protein primate diet (Envigo, Madison, WI) supplemented with fresh fruit and vegetables, with water available ad libitum. Animal care and veterinary staff monitored the monkeys’ health status twice daily. The 16 macaques were randomly assigned to four groups (n = 4 per group): Saline, SalineSIV, Morphine, and MorphineSIV. Animals in the Morphine and MorphineSIV groups received intramuscular morphine injections, gradually increased over two weeks to a maintenance dose of 6 mg/kg administered twice daily on weekdays and once daily on weekends. This dose was maintained for seven weeks before viral inoculation. Animals in the Saline group received saline injections on the same schedule. Macaques in the Saline SIV and Morphine SIV groups were intravenously inoculated with 200 TCID_50_ of SIVmac251 (viral stock was obtained from the Tulane National Primate Research Center). Five weeks post-inoculation, all SIV-infected animals began receiving a daily subcutaneous injection of antiretroviral therapy (ART) at 1 mL/kg, containing 40 mg/mL emtricitabine (FTC), 20 mg/mL tenofovir (TFV), and 2.5 mg/mL dolutegravir (DTG), which continued until necropsy. The three ARTs were dissolved in a vehicle made up of 15% Kleptose HPB (Roquette, parenteral grade) (wt/vol) in 0.1 N NaOH, adjusted to a final pH of 7.4.

### Viral load in plasma and CSF

Peripheral blood was collected from the femoral vein and CSF was obtained via direct puncture of the cisterna magna or lumbar puncture at various time points during the study. All sample collections were performed under anesthesia using either ketamine-HCl (5–20 mg/kg) or a combination of tiletamine and zolazepam (Telazol; 3–5 mg/kg) to facilitate monitoring of SIV viral loads. SIV RNA concentrations in plasma and CSF were quantified by quantitative reverse transcription PCR (qRT-PCR) as previously reported^68,69^. Briefly, blood was drawn into K2-EDTA vacutainer tubes (Becton, Dickinson, San Diego, CA, USA) and plasma was separated within 4 hours of collection. Viral RNA was extracted from 140 μL of plasma and CSF using the QIAamp Viral RNA Mini Kit (Qiagen, Germantown, MD, USA; cat. no. 52906) following the manufacturer’s protocol. Quantification of SIV *gag* RNA was performed using the TaqMan RNA-to-Ct 1-Step Kit (Thermo Fisher Scientific, MA, USA; cat. no. 4392938) on an Applied Biosystems QuantStudio 3 real-time PCR system (Applied Biosystems, Waltham, MA, USA). The primers and probe used for *gag* RNA detection included **forward primer (SIVGAGF):** 5′-GTCTGCGTCATCTGGTGCATTC-3′; **reverse primer (SIVGAGR):** 5′-CACTAGGTGTCTCTGCACTATCTGTTTTG-3′; **probe (SIVP):** 5′-/6-FAM/CTTCCTCAG/ZEN/TGTGTTTCACTTTCTCTTCTGCG/3IABkFQ/-3′.

### Isolation of myeloid cells in the brain

The necropsy was performed when each infected animal’s plasma viral load was under the detection limit for a minimum of 24 weeks. Uninfected animals were necropsied per approved animal protocol after an equivalent time span of saline or morphine administration. Animals were deeply anesthetized with ketamine plus xylazine, and blood was cleared from the brain and other organs by intracardial perfusion with sterile PBS containing 1 U/ml heparin. Brains were harvested, and approximately half of the brain was taken for microglia/macrophage isolation. Microglia/macrophage-enriched cellular isolation was performed using our previously described procedure^70^. After isolation, the cells were resuspended in 10% DMSO and 90% fetal bovine serum and subjected to slow controlled freezing followed by storage in liquid N2.

### Single nuclei preparation and multiomic sequencing (ATAC and gene expression)

Cryopreserved cell isolates were rapidly thawed in a 37° C water bath. The cell recovery procedures were well described in our previous publications^70^. After the recovery, cells were washed and counted by Coulter Counter Z1. Once cell concentration was known, cells were transferred to ice-cold PBS and stained with CD11b (Biolegend 101257) and UV-blue live/dead (Invitrogen L23105). Cells were washed, resuspended in flow cytometry staining buffer (e-bioscience), and sorted on an Aria2 flow cytometer (BD Biosciences, San Jose, CA, USA).

Nuclei were isolated from microglia cells using the 10XGenomics protocol. Nuclei were then quantified on a hemocytometer and concentrated to approximately 2200–2400 nuclei per μL. Based on 10× Genomics parameters targeting 8000 nuclei, the ideal volume of cells was loaded onto the 10× Genomics (Pleasanton, CA, USA) Chromium Next GEM Chip J and placed into the Chromium Controller for nuclei capturing and library preparation. The prepared libraries were sequenced using Illumina (San Diego, CA, USA) Novaseq6000 sequencers. The sequences have been deposited in NCBI GEO (accession number GSE297853).

### Pre-processing of single nuclei multiomic data

Sequenced samples from 16 rhesus macaques were processed using the 10× Genomics CellRanger ARC pipelines (version: 2.0.2). The multiomic data was demultiplexed and aligned to a customized genome combining the rhesus monkey reference genome (NCBI RefSeq assembly Mmul_10) and an SIV genome that we constructed by sequencing our virus stock (NCBI GenBank, accession number PP236443). We used the optimized method (i.e., SIV genome sequence with only one LTR kept) for mapping SIV DNA fragments and RNA transcripts, but we still did not identify a single SIV-positive cell for all 16 samples. The counting summary statistics generated by 10x Genomics for each sample are shown in **Supplementary Table 9**. The downstream analyses were implemented with R (version 4.3) and Python (version 3.12.7).

### Characterization of cell phenotypes for single nuclei mutiomics data

Pre-processed ATAC-seq data from multiomic sequencing processed with CellRanger arc were read with the ArchR R package (version: 1.0.2)^71^. We built our own gene and genome annotation used in ArchR by the BsgeomeForge R package and the GenomicFeature R package. After successfully reading the fragment files, we performed several steps of quality controls for the cell barcodes in the fragment files. Firstly, the cell barcodes with less than 1000 fragments per cell and a TSS enrichment score of less than four were removed. Then, the cell barcodes with only RNA data or ATAC-seq data were removed. Finally, the cell barcodes with gene count and UMI count of less than 400 and mitochondria percentage of more than 15% were removed. Finally, we identified and removed the inferred doublets. After filtering, 106,130 cells were left for downstream analyses. The feature barcode matrices from snRNA-seq data were imported in the Seurat R package (version: 4.4.0)^72^, and only the cell barcodes passed through the aforementioned QC parameters were kept. The general Seurat workflow was then performed, including the NormalizeData, FindVariableFeatures, ScaleData, and RunPCA to normalize the data and reduce the dimensionality. The data normalization was using a natural logarithm with a scale factor set as 1e6. The top 2000 most variable genes were scaled and used for PCA. The batch effects were then removed with Seurat’s implementation of Harmony. Then, we ran the UMAP using the RunUMAP function and clustered the cells using the FindNeighbors and FindClusters functions with the first 30 PCs and 0.3 as resolution. After screening the normalized RNA expression of the gene markers for microglia (e.g. P2RY12, GPR34, CX3CR1), CNS-associated macrophages (e.g. MAMU-DRA, MAMU-DRB1, MAMU-DRB5), and lymphocytes (e.g. CD3D, CD3E, GZMB), we removed lymphocyte population.

The remaining 105,818 brain myeloid cells were then reclustered with the same Seurat functions and parameters, generating nine different cell clusters. Characterizing the cell cluster at the RNA level was achieved using the FindAllMarkers function in Seurat (parameters: test. use = “Wilcox”, logfc. threshold = 0.25, only. pos = T, min. pct = 0.25), followed by GO analysis implemented through cluster Profiler (version: 4.0.2)^73^. We also identified the cell markers using snATAC-seq data, which was achieved by the getMarkerFeatures function in ArchR (parameters: testMethod = “Wilcoxon”)) and the getMarkers function (cut-off was set as FDR ≤ 0.05 and log_2_FC ≥ 0.25) using the gene score matrix predicted by ArchR.

### Cell composition analysis

Statistical testing of cell type composition differences between each pairwise group comparison among the four experimental groups was conducted using the speckle package’s implementation of the propeller method^37^. Propeller applies an empirical Bayes moderated t-test to assess differences in the proportions of myeloid cell clusters between groups. Resulting raw p-values were adjusted for multiple testing using the Benjamini-Hochberg false discovery rate (FDR) correction. A summary of the statistical results is provided in **Supplementary Table 3**.

### Differentially expressed gene analysis between treatment groups

The cells from each cell cluster were subset for comparisons between two treatment groups. The parameters used for testing were set the as same above and the cut-off was set as FDR ≤ 0.05 and log_2_FC ≥ 0.5.

DEG analysis on pseudobulk data was performed using the muscat R package (version 1.16)^74^. Single-cell data were aggregated by clusters and samples using the aggregate data function in muscat for pseudobulk analysis. Differential expression analysis was conducted using muscat implementation of DESeq2^75^ with contrast information for two treatment groups. Genes with locally adjusted p-value < 0.05 and an absolute log_2_FC > 0.25 were considered significantly differentially expressed.

### Principal Component Analysis (PCA) on pseudobulk data

We first performed PCA on all brain myeloid cells, then subset the key brain microglia/CAM cell clusters (i.e. Homeostatic, Activated, Antiviral microglia and CD16+CAM) for analysis. We aggregated the single-cell expression for all brain myeloid cells or the cells in individual cluster by the AggregateExpression function in the Seurat. The PCA was conducted on the normalized, scaled, and centered RNA expression data of top 5000 highly variable genes using the prcomp function in the R stats package. The coordinates of samples and selected variables on the first two PCs were plotted using the ggplot2 package. The Euclidean distance was then calculated between each experimental group for each cluster using the dist function in the R stats package.

### Single-cell level weighted gene co-expression network analysis (scWGCNA)

The WGCNA at the single-cell level was performed using the hdWGCNA R package.^43^ Data preparation for WGCNA was carried out using the SetupWGCNA function, retaining genes expressed in at least 5% of cells. Then the cells were grouped by cell cluster and sample to construct a metacell matrix, with the max_shared parameter (allowable overlap between metacells) set to 10. After normalizing the constructed metacell matrix, the TestSoftPowers function was used to determine the optimal soft-power threshold, which was subsequently applied in the ConstructNetwork function to build the co-expression network. We then computed the harmonized module eigengenes (hMEs) for individual cells using the ModuleEigengenes function. For each gene, eigengene-based connectivity (kME) was calculated using the ModuleConnectivity function. Hub genes identified in modules 2, 4, 5, 6, and 8 were subsequently subjected to Ingenuity Pathway Analysis (IPA), based on their kME values. Only signaling pathways were considered, and the results were filtered using a threshold of −log(p-value) > 1.3 and z-score > 2.

To investigate the association between identified modules and morphine use/infection, module-trait correlation analysis was performed on both individual clusters and all brain myeloid cells. This analysis was conducted using the ModuleTraitCorrelation function with the group.by the parameter set to cluster information. For correlation between ART-treated infection and modules, the cells in Saline SIV and Morphine SIV groups were set as positive (“1”) and the other two groups were set as negative (“0”). Similarly, for the correlation between morphine use and modules, the cells in Morphine and Morphine SIV groups were set as positive, and other groups were set as negative.

Differential module eigengene (DME) analysis was performed to identify changes between treatment groups using the FindDMEs function, with the Wilcoxon rank-sum test as the statistical method. Pairwise comparisons included Saline SIV vs. Saline, Morphine SIV vs. Saline, Morphine vs. Saline, Morphine SIV vs. Morphine, Morphine SIV vs. Saline SIV, and SalineSIV vs. Morphine. The DME results were visualized using the PlotDEMsLollipop function, which displays the fold-change for each module, with dot size representing the number of genes in the module. Modules that reached statistical significance were labeled with an “X”.

Module preservation test was performed between macaque and human data. The modules identified in macaque data was first projected to the data from cART-treated human samples through the Project Modules function. Then, the module preservation test was performed by ModulePreservation function, the n_permutations was set to 150. The summary statistics, including individual preservation and quality statistics, were used for plotting.

### Peak Calling and transcription factor (TF) motif enrichment

Single-cell chromatin accessibility data were used to generate the pseudobulk replicates by the ArchR function, addGroupCoverages, for peak calling with MACS2^76^. The implementation of MACS2 in the ArchR was through the addReproduciblePeakSet function, in which we set genome size as 2.2e9, which, as suggested by MACS2 documentation, is 78% of the total genome size of rhesus macaques.^77^ The pseudobulk replicates and peak calling were based on all myeloid cell clusters identified in this study. Then, we determined the enrichment of transcription factor binding sites for the marker peaks by the peakAnnoEnrichment function. Before the enrichment, we used JASPAR 2020 database to annotate the peaks, which was followed by the chromVAR R package^78^ embedded in ArchR to calculate the TF enrichment on a per-cell basis. A background peak set controlling for total accessibility, and GC-content was generated by the addBgdPeaks function before ChromVAR was run with the addDeviationsMatix function, using the JASPAR motif set to calculate enrichment of chromatin accessibility at different TF motif sequences in a single cell.

### Human-macaque scRNA-seq data integration and analysis

We evaluated the performance of four batch effect correction methods and one cross-species integration method for integrating scRNA-seq data of microglia from human and rhesus macaque samples. The batch correction methods included Seurat CCA, scVI, scANVI, and scGen, while SATURN was used as the cross-species integration method. Since batch effect correction methods require a consistent gene symbol convention across species, we tested three orthologous gene mapping approaches to assess their impact on integration performance. Specifically, we utilized the HomoloGene database via the orthogene R package (https://github.com/neurogenomics/orthogene), gene ortholog mappings from the GeneOrthology R package (https://github.com/AllenInstitute/GeneOrthology), and a custom gene conversion database. The custom database excluded non-protein-coding genes, mitochondrial genes, and genes with one-to-many or many-to-many ortholog mappings, retaining only one-to-one orthologs between human and macaque.

Gene symbols in the rhesus macaque cell count matrices were converted based on each of these databases, and only genes shared between the human and macaque datasets were retained for integration. We tested the integration methods using scRNA-seq data from three human samples^59^ and six rhesus macaque samples^33^. Quality control was performed separately on human and macaque datasets using species-specific thresholds **(Extended Data Fig. 4a)**, and only microglia were retained for downstream analysis. Prior to integration, we performed clustering and broad classification of microglia within each species. Integration methods were implemented according to the developers’ recommended workflows. For batch effect correction, each sample (human or macaque) was treated as a separate batch. For scGen, scANVI, and SATURN, cell annotations derived from our broad classification were also incorporated into the integration process. To benchmark the integration methods, we used the corrected low-dimensional embeddings: corrected principal components for Seurat CCA and SATURN, and the corrected latent representations for scGen, scVI, and scANVI. Benchmarking metrics were calculated using the scib package^58^.

To incorporate a larger number of microglia across species, we further applied scGen to integrate scRNA-seq data from all 16 animals in our study along with publicly available datasets from GEO: GSE204702^60^ and GSE221688^59^ (human), GSE253835^31^ and GSE195574^33^ (rhesus macaque). For cross-species gene name conversion, we used the custom database described above and followed the same integration workflow. The resulting scGen-corrected count matrix was used for clustering and UMAP visualization using Scanpy (version 1.11.1)^79^. To characterize each cluster, we calculated homeostatic scores based on the expression of P2RY12, GPR34, CX3CR1, SALL1, and TMEM119. Additionally, the activated score was calculated based on the expression of CCL4, CCL3, IL1B, C1QB, C1QC, HLA-DRA, CD74, HLA-DMB, HLA-DPA1, and CD86, the antiviral score was calculated based on the expression of genes (i.e. MX1, IFI16, ADAR, TRIM22, IFIT2, MX2, DDX60, HERC5, SAMHD1, UNC93B1) enriched in virus-related and antiviral pathways **(Extended Data Fig. 1f)**, the preactivated score was calculated based on the expression of CD83, TNF, FOS, FOSB, JUN, JUNB, and the proliferating score was calculated based on the expression of MKI67 and TOP2A. Those scores were calculated by the sc.tl.score_genes function in scanpy. The low-activated microglial clusters (i.e. clusters 1 and 12) were defined as clusters with a similar homeostatic and activated score, and the mid-activated microglial clusters (i.e. clusters 3, 5, and 10) were defined as clusters with relatively higher activated scores compared to homeostatic score, and the hyper-activated microglial clusters (i.e. cluster 8 and 9) were defined as clusters with the neglectable homeostatic score but high activated score.

### Statistics

DEG analysis at the single-cell level between treatment groups or between cell clusters was conducted using the non-parametric Wilcoxon rank-sum test, with p-values adjusted via the Benjamini-Hochberg method. For transcriptomic data, statistical significance was defined as an FDR ≤ 0.05 and a log_2_FC ≥ 0.5. For transcription factor deviation scores, significance was defined as FDR ≤ 0.05 with a mean difference ≥ 0.5 between treatment groups or ≥ 1 between clusters. Sample-level DEG analysis between treatment groups was performed using DESeq2, and genes with an adjusted p-value ≤ 0.05 and log_2_FC ≥ 0.25 were considered significant. Comparisons of cell-type composition between treatment groups were assessed using empirical Bayes moderated t-test with Benjamini-Hochberg method for p-value adjustment.

## Supplementary Material

Supplementary Files

This is a list of supplementary fi les associated with this preprint. Click to download.

• SupplementaryTable3Statisticsforcelltypecompositionbetweentreatments.xlsx

• SupplementaryTable4.CelllevelDEGsbetweentreatmentgroupsandcontrolgroup.xlsx

• SupplementaryTable1.DEGanalysisforstudyingbrainmyeloidcellsinmorphineandcARTtreatedSIVinfection.xlsx

• SupplementaryTable5.Pseudobulkcomparisonsbetweentreatmentgroups.xlsx

• SupplementaryTable9.Animalinformationandseqsummary.xlsx

• SupplementaryTable7.Upregulatedpathwaysforselectedmodules.xlsx

• SupplementaryTable6.Hubgenesforthegenemodulesidentifi edbyWGCNA.xlsx

• SupplementaryTable2.GOBPanalysisforDEGsineachcluster.xls

• SupplementaryTable8.Humanandmacaquedatasetforintegration.xlsx

• EXTENDEDDATA.docx

## Data Availability

Multiomic (snRNA-seq + snATAC-seq) data from 16 animals have been deposited with full public access in the NCBI GEO repository with accession number GSE297853. For integrating human and macaque scRNA-seq data for analysis, we downloaded the fastq files from the GEO repositories with accession numbers GSE204702 and GSE221688, GSE253835 and GSE195574.

## Code availability

The code used for analyzing the data in this study was deposited in this GitHub repository: https://github.com/Howard-Fox-Lab/SCORCH_Multiomics.
